# Applications of Adductomics in Chemically Induced Adverse Outcomes and Major Emphasis on DNA Adductomics: A Pathbreaking Tool in Biomedical Research

**DOI:** 10.3390/ijms221810141

**Published:** 2021-09-20

**Authors:** Tapan Behl, Mahesh Rachamalla, Agnieszka Najda, Aayush Sehgal, Sukhbir Singh, Neelam Sharma, Saurabh Bhatia, Ahmed Al-Harrasi, Sridevi Chigurupati, Celia Vargas-De-La-Cruz, Yahya Hasan Hobani, Syam Mohan, Amit Goyal, Taruna Katyal, Ewa Solarska, Simona Bungau

**Affiliations:** 1 Chitkara College of Pharmacy, Chitkara University, Rajpura, Punjab 140401, India; tapanbehl31@gmail.com (T.B.); aayushsehgal00@gmail.com (A.S.); sukhbir.singh@chitkara.edu.in (S.S.); neelam.mdu@gmail.com (N.S.); 2 Department of Biology, University of Saskatchewan, 112 Science Place, Saskatoon, SK S7N 5E2, Canada; maheshgupta65@gmail.com; 3 Department of Vegetable Crops and Medicinal Plants, University of Life Sciences in Lublin, 20-950 Lublin, Poland; 4 Natural & Medical Sciences Research Centre, University of Nizwa, Birkat Al Mauz, Nizwa 33, Oman; sbsaurabhbhatia@gmail.com (S.B.); aharrasi@unizwa.edu.om (A.A.-H.); 5 Department of Medicinal Chemistry and Pharmacognosy, College of Pharmacy, Qassim University, Buraydah 52571, Saudi Arabia; sridevi.phd@gmail.com; 6 Faculty of Pharmacy and Biochemistry, Academic Department of Pharmacology, Bromatology and Toxicology, Centro Latinoamericano de Enseñanza e Investigación en Bacteriología Alimentaria, Universidad Nacional Mayor de San Marcos, Lima 15001, Peru; cvargasd@unmsm.edu.pe; 7 E-Health Research Center, Universidad de Ciencias y Humanidades, Lima 15001, Peru; 8 Department of Medical Laboratory Technology, Faculty of Applied Medical Sciences, Jazan University, Jazan 114, Saudi Arabia; yahobani@jazanu.edu.sa; 9 Substance Abuse and Toxicology Research Center, Jazan University, Jazan 114, Saudi Arabia; syammohanm@yahoo.com; 10 GHG Khalsa College of Pharmacy, Gurusar Sadhar, Ludhiana 141104, India; goyal141186@yahoo.com; 11 RBMCH Division, ICMR Head Quarters, Ramalingaswami Bhawan, Ansari Nagar, New Delhi 110029, India; tarunakatyal@gmail.com; 12 Department of Biotechnology, Microbiology and Human Nutrition, Faculty of Food Science and Biotechnology, University of Life Sciences in Lublin, 8 Skromna Street, 20-704 Lublin, Poland; ewa.solarska@up.lublin.pl; 13 Department of Pharmacy, Faculty of Medicine and Pharmacy, University of Oradea, 410073 Oradea, Romania; simonabungau@gmail.com

**Keywords:** adductomics, DNA adduct, protein adduct, cancer detection tool

## Abstract

Adductomics novel and emerging discipline in the toxicological research emphasizes on adducts formed by reactive chemical agents with biological molecules in living organisms. Development in analytical methods propelled the application and utility of adductomics in interdisciplinary sciences. This review endeavors to add a new dimension where comprehensive insights into diverse applications of adductomics in addressing some of society’s pressing challenges are provided. Also focuses on diverse applications of adductomics include: forecasting risk of chronic diseases triggered by reactive agents and predicting carcinogenesis induced by tobacco smoking; assessing chemical agents’ toxicity and supplementing genotoxicity studies; designing personalized medication and precision treatment in cancer chemotherapy; appraising environmental quality or extent of pollution using biological systems; crafting tools and techniques for diagnosis of diseases and detecting food contaminants; furnishing exposure profile of the individual to electrophiles; and assisting regulatory agencies in risk assessment of reactive chemical agents. Characterizing adducts that are present in extremely low concentrations is an exigent task and more over absence of dedicated database to identify adducts is further exacerbating the problem of adduct diagnosis. In addition, there is scope of improvement in sample preparation methods and data processing software and algorithms for accurate assessment of adducts.

## 1. Introduction

Adductomics has become most prominent technique in recent times, and it is one of the rapidly emerging disciplines with the potential to dramatically transform the landscape of toxicological research. The time has probably come for adductomics to join the elite club of words such as genomics, proteomics, and metabolomics. Though the term adductomics first appeared in a journal published in 2006 [1] and currently its applications reached almost all areas in toxicological research. Adductomics is a transformational biomedical research tool which utilizes “omics” approach to characterize and quantify exogenous and endogenous reactive compounds, to which the individual is exposed; leveraging compound-specific adducts biomarkers. Chemical exposure is generally driven by various factors such as environmental, genetic, and lifestyle, were characterized by high-level inter-person variability and incorporates a lifetime component, making it unique to every individual.

**Figure 1:**
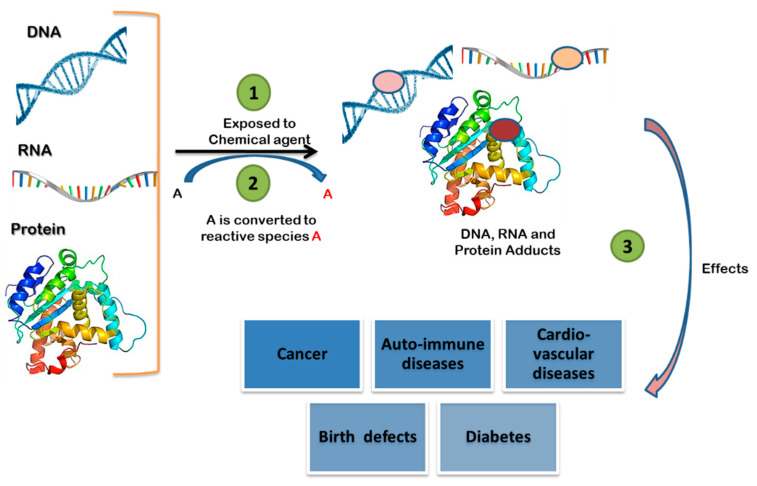
Schematic representation of how chemical agents reacts with DNA, RNA and proteins in cells and leading formation of respective adducts, followed by their resulting in toxicological outcomes. In the figure ① indicating the exposure to toxicant ② at cellular level due to enzymatic activity production reactive species or metabolites which are highly reactive than parent molecule ③ generated reactive molecules interact with various cellular components such as protein, RNA and DNA and leading to cause of various pathological outcomes.

Adductomics majorly focuses on investigation of adducts formed from covalent modification which are in irreversible in nature with bio-macromolecules by exogenous or endogenous reactive electrophile compounds. Reactive compounds interact with nucleophilic hot spots (susceptible sites to electrophiles) present in DNA, lipids, proteins, RNA, and other macromolecules leading formation of adducts (as shown in Figure 1). Biomonitoring of reactive metabolites regardless of their origin, whether exogenous or endogenous, is challenging due to their short-life in vivo and adductomics provided unique opportunity to detect covalent adducts that are relatively stable and long-lived. Adductomics studies broadly uses two approaches (1) targeted and (2) untargeted; former method focuses on the detection of specific adducts upon exposure to a specific chemical agents, and later category aims to characterization of total adducts through covalent bonding [2,3].

In some instances, the chemical agents in natural form do not directly bind with the biological molecules to form adducts, however, transformation to reactive metabolites occurs by metabolic enzymes like Cytochrome P450 systems and formed reactive metabolites can bind with protein, RNA and DNA [4,5]. Reactive electrophiles generated from genotoxicants covalent bonding with DNA can occur through several mechanisms (1) arylamination [6], (2) alkylation [7], (3) bis-electrophile cross-link development [8], and (4) adducts with highly reactive intermediates produced due to lipid peroxidation [9,10,11] or reactive oxygen species. The type and nature DNA adducts formed is typically dependent on multiple factors such as the chemical structure of the reactive chemicals, capacity of the chemical to intercalate with DNA, and the nature of the electrophiles. Evidences from aflatoxin, tobacco-specific nitrosamine (NNK), polycyclic aromatic hydrocarbon (B[a]P), heterocyclic aromatic amine and other exogenous toxicants form a different type of DNA adducts owing to their different structural and chemical properties [12]. Adduct formation can cause significant impact on biological system and leading to deleterious health complications [13], such as diabetes, neurodegenerative diseases, autoimmune diseases, cancer, birth defects [14], and cardio-vascular diseases [15]. Understanding chemical induced adducts using adductomics can be indispensable to gain better insights in these diseases and provides novel insights in effective drug development. Adductomics can have diverse practical applications across diverse domains are evident in its role (1) prognosis of diseases, (2) environment health assessment [14], (3) development of personalized and precision medicine [16], (4) detection of biomarkers for various chemical exposures [17,18,19]. Measurement of DNA adducts formed upon exposure to a potential carcinogen in target organs is one of primary methods to evaluate the genotoxic capability of a chemical compound, and it serves as most sophisticated method to determine genotoxicity potential of chemical. Besides, adductomics also identifies underlying risk factors of pathogenesis and underlying molecular mechanisms of chemical induced toxicities. Data from adductomics would also serve as a guide for regulatory agencies and empowers other stakeholders in taking preventive measures against the toxic chemical’s exposure.

**Figure 2:**
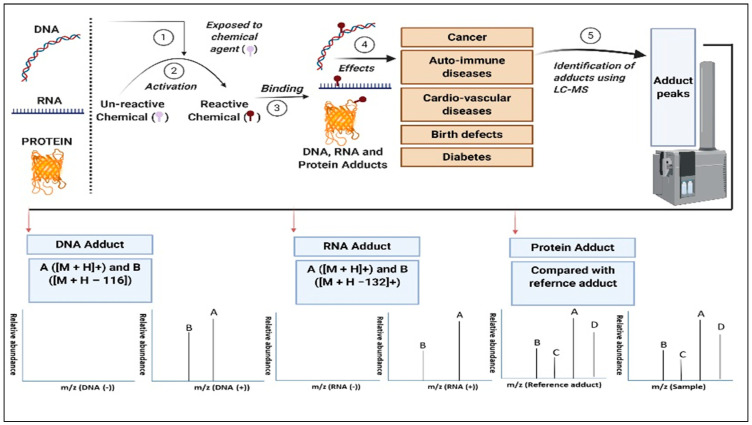
Three types of adducts are formed in biological systems (or organisms) when exposed to chemical agents and their associated health hazards. The adducts (A, B, C, and D) are identified using a fragmentation pattern in the LC-MS spectra. In DNA adducts, the characteristic peaks from the protonation events (M + H) + and cleavage events (M + H−116) + are detected. In RNA adducts, the characteristic peaks are detected from the protonation (M + H)+ and cleavage events (M + H−132)+ [20,21]. To identify the protein adducts, we generally compare the spectra data of unknown compounds with the reference adduct. Reference adducts would be synthesized by assuming an electrophile, and they should be compared with the unknown adducts of interest. By adding proposed precursor electrophiles to plasma or whole blood/lysate the reference adducts can be generated, and they are subjected to fragmentation using LC-MS. The synthetic adducts then further will be compared with the novel or unidentified adducts with m/z of the precursor ions, also studying fragmentation patterns and retention times. On the other hand, by using UNIMOD database that lists the protein modifications is also used to identify the adducts [22].

Rapid improvement in methods and tools in identify and quantify adducts have transformed adductomics as one of the most promising disciplines in toxicology. Collection of tissue samples and sample preparation is one of key factor in detection of various adducts. Recent scientific advancements and increased precision in detection methods as well as sample preparation methods allow to collect the samples in non-invasive sampling and use of body fluids (blood plasma or serum, and urine) so-called liquid biopsy. Use of non-invasive method such as liquid sampling provided various advantages like 1) samples can be collected at various intervals without causing much discomfort to patients 2) easy of collection and storage 3) ease of transportation. Numerous tools that are currently being used to diagnose the adducts in the biological systems are 32P-Postlabeling, fluorescence, immunoassay, electrochemical detection, and Mass Spectrometry (MS) (LC-MS, GC-MS, CE-MS) [20]. From among the tools, high-resolution Mass Spectrometry (HRMS) is the widely used and suitable method or assessing qualitative and quantitative adduct formation, including the identification of the covalent conjugate sites within bio nucleophiles. Fragmentation pattern in Mass Spectrometry is used in identification of different types adducts and noteworthy aspect regarding the DNA and RNA adducts is the near-universal loss of ribose and deoxyribose from the parent molecule giving characteristic peaks at (M + H−116)+ and (M + H−132)+ respectively [20,21]. On the other hand, unknown protein adducts are identified by comparing the test adducts spectral data with the reference adduct (Figure 2). Firstly, reference adducts should be synthesized by assuming a particular electrophile, and then they will be matched with the novel adducts of interest to study further. By adding proposed precursor electrophiles to plasma or whole blood/lysate the reference adducts can be generated, and they are subjected to fragmentation using LC-MS. The synthetic adducts then further will be compared with the novel or unidentified adducts with m/z of the precursor ions, also studying fragmentation patterns and retention times. Moreover, this approach also contributes to generating an extensive database of the reference protein adducts by which identification of unknown protein adducts becomes much easier [22].

## 2. Application of Adductomics

It is an indisputable fact that adductomics has an influential role in unleashing new insights in epidemiology, etiology, pathology, and hazard and risk assessment. Detailed illustration of applications of adductomics is discussed in detail.

### 2.1. Adductomics and Disease

In current world exposure to chemical agents from the diet, environmental pollutants, and drugs has become prevalent and resulting in causing harmful health outcomes. Investigating exposure-related adverse health outcomes and their underlying mechanisms are essential to understand, adductomics can be better alternative in current times.

Recent studies suggest that exposure to environmental toxicants plays crucial role in pathogenesis and progression of chronic inflammatory diseases and non-communicable diseases (neurological disorders, auto immune diseases, lung diseases, cardiovascular complications cancers and various cancers), and one of key mechanism is found to be the formation of adducts. Chronic diseases are the product of genetic factors (G) and exposures (E) and their associated interactions (GxE) [3,22]. According to data of the World Health Organization (WHO) it is estimated that nearly half of the mortality is result of exposure to toxic chemicals, which includes particulate air pollution (comprising indoor air pollution and occupational exposure—14%), active and passive smoking (13%), Increased levels of sodium in plasma (6%), and consumption of alcohol (5%) [23]. Epidemiologic evidence suggests that even in populations with stable genetic makeup across generations and with migration, are experiencing variations in cancer incidence that plausibly showcases changing exposures and role of exposure in etiology [23,24,25]. Wang et al., [26], using a data-driven approach by performing Exposome Wide Association Studies (EWAS), identified 18 chemical structural groups among 2000 detected adducts, which were associated with cardiovascular disease in samples containing 75 exposed and 75 control population. Trimethylamine-N-oxide (TMAO) is a product of human and microbial combined metabolism product of choline; positive correlation observed between increased plasma TMAO levels and increased cardiovascular disorders risk which signify the connection of endogenous electrophiles and gut microbiome in pathogenesis. Similarly, Bae et al. [27] also found correlation between increased TMAO plasma levels and occurrence of colorectal cancer, also suggesting role of gut microbiota participation. In another study, interactions between Cys-34 and reactive oxygen species (ROS), forming adducts was investigated by using LC-MS, and prolonged exposure to these ROS is perceived to be associated with chronic diseases; and oxidation products (adducts). Results from these studies can also serve as key for identification of biomarkers of ROS exposure [28]. All the above shreds of evidence convey those exposures are significant determinants of diseases and operate in addition to variations in genetic background. Growing evidence of adductomics and their roles in human disease pathology render us to deciphering the etiology so that necessary precautionary measures can be taken to address them.

### 2.2. Adductomics and Pollution

Adductomics finds application in pollution assessment and provides information pertaining to their toxic effects on biological systems signaling status of environment’s health. For example, Polycyclic aromatic hydrocarbons (PAHs), toxic pollutants, are a group of structurally similar hydrocarbons released into the atmosphere due to incomplete burning of organic matter, tobacco smoke, urban air pollution, and automobile exhaust emissions [29]. The PAHs can form adducts with DNA via reactive intermediates when activated using Cytochrome P-450 systems, making them highly carcinogenic [30]. One such electrophilic reactive species formed by CYP 1A1 and CYP 1B1 is PAH-dihydro-diol epoxide, which can react with exocyclic groups present in nucleotides like guanine, adenine, and cytosine present in DNA [31]. Similarly, multiple PAH-DNA adducts are formed with other reactive intermediates in individuals who are exposed to PAHs, and DNA adducts formed are studied using 32P-Postlabeling and LC-MS [30]. Simultaneous assessment of the entire pool of PAH-DNA adducts in individuals equips us with a comprehensive exposure profile and facilitates a better understanding of the underlying mechanistic pathways [32]. Another study established the relationship between formation of PAH-DNA adducts at ambient air pollution in exposed mothers and newborns in Poland, which is evident in the dose-response curve that manifested a proportionate increase in the number of DNA adducts with the extent of air pollution [33]. In the Mediterranean population, bulky DNA adducts are correlated with environment ozone pollution that contributes to photochemical smog [34]. Hylland et al. [35] used DNA adducts as signature biomarker to examine the extent of pollution at various locations in the Northeast Atlantic region near to coastal and offshore. Adduct as a biomarker (DNA adduct) alerts risk exposure by providing early warning information and assisting in improving hazard assessment for aquatic organisms and ecological risk assessment [36]. Also, it was revealed that DNA adducts (PAH-DNA adducts) would also help determine a biologically effective dose of PAH exposure, furnishing the presence and extent of environmental pollution and its association with the development of cancer. PAHs are ubiquitous, and their presence in oil and gas mixtures results in contamination of the aquatic ecosystem during oil and gas exploration. Detection of PAH-DNA adducts can also use as potential biomarkers of environmental contamination and genotoxicity studies in aquatic organisms [37]. Moreover, several reports presented evidence for the impact of crude oil and producer gas on formation of DNA adducts in marine organisms in both laboratory animals and in vivo after major oil spills [38,39,40].

One of the most exposed PAH is benzo[a]pyrene (BaP) and it is also the most studied and measured substance. However, BaP does require enzymatic activation to become genotoxic metabolite whose activity can be modulated by cytochrome P450 oxidoreductase (POR) enzyme. In recent in vitro and in vivo studies conducted using knock out of POR enzyme results ted in increased adduct formation. A significant increase in the BaP-DNA adduct was observed in wild type mice in which POR was specifically deleted in hepatocytes [41]. In another study human hepatoma HepG2 cells carrying a knockout (KO) in the POR gene as a humanin vitro model and treatment with BaP for 48 h caused similar cytotoxicity as seen in KO mice study [42]. Collectively, these new finding suggest that CYPs plays important role in BaP metabolism as well as DNA adduct formation. However, investigations need to be done to further understand the key role of various CYP enzymes in modulating or moderating toxicities of chemicals.

### 2.3. Adductomics in Precision Medicine in Cancer

Traditionally, DNA modifying drugs (drugs reacting covalently with DNA or drugs forming cross-links with double strand) are the first line of therapy to treat cancer, but the emergence of resistance, unresponsiveness of patient and detrimental side-effects associated makes them very concerning to use. Owing to the huge toxicity of traditional anticancer drugs, precision in treatment holds great significance to reduce toxic side-effects and increase efficacy, and this is accomplished by designing drug-based biomarkers (Drug-DNA biomarkers), which could yield appropriateness of drug to which patient might respond [16]. This biomarker-driven drug selection and patient stratification play a significant role in discovering and developing new cancer drugs, and better targeting of traditional chemotherapeutic drugs; designing such biomarkers requires adductomics, which identify and quantify adducts formed due to anticancer drugs. Biomarkers can become handy for clinicians to better target the medication; drug efficacy predictability, resistance, toxicity, response in patients, and stratification based on their response [43]. Detecting drug-DNA adducts could also be a predictive biomarker for cancer drug induced DNA damage, to determine drug induced DNA damage there are three major exposure approaches are used. Firstly, upon first treatment with chemotherapeutic agents in patients, analysis for detecting adducts in various biological samples such as circulating tumor cells, tumor tissue biopsy and other tissues at therapeutic levels of chemotherapy. Secondly, patients will be injected with micro doses of DNA alkylating drugs and look for adduct formation in tumor tissue biopsy and peripheral blood mononuclear cells (PBMC). Finally, cancer cell and normal cells are exposed to DNA modifying agent’s ex vivo to see if there is any adducts are formed.

**Figure 3:**
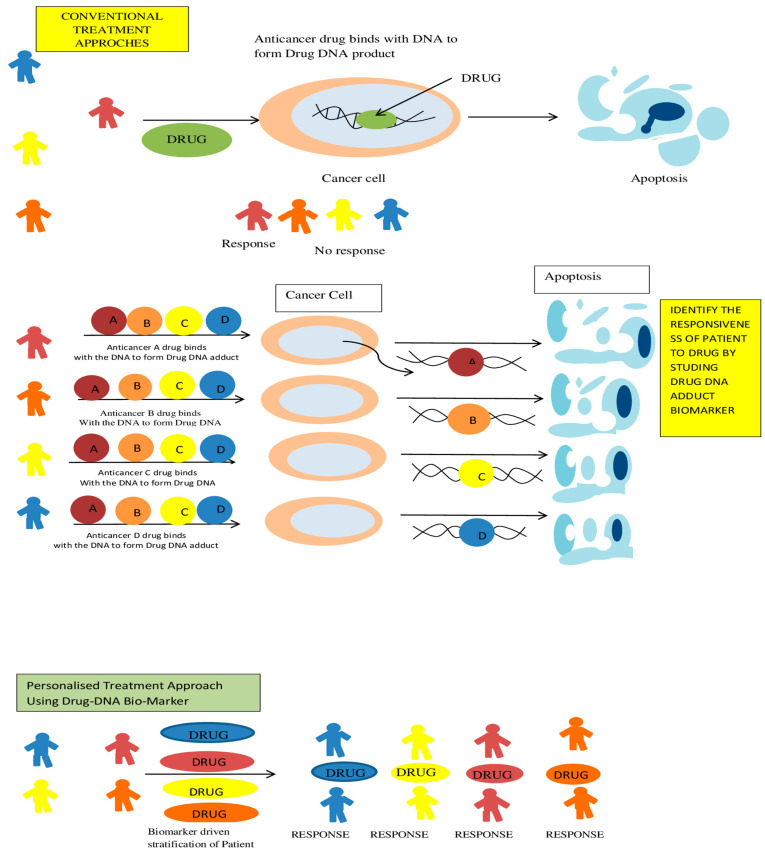
Application of adductomics in precision medicine of anticancer drugs for better targeting and reducing the toxicity.

Leveraging any one of the approaches mentioned above help in evaluating the binding capability of the drug to the DNA, and if drug binds then medication should be continued or else resort to other drugs; this evaluation process is repeated till the desired drug that forms an adduct with DNA, eventually accomplishing desired anticancer effect. To further potentiate above results, similarly there was positive correlation was observed in preclinical and clinical data for Drug induced DNA adduct and physiological response. In the study following classes of anticancer agents were studied which are platinum-based drugs, nitrogen mustards, reductase activated drugs, minor groove binding drugs and hypoxia activated drugs [44]. This positive correlation witnessed in the majority of the studies demonstrates the high potential of DNA adductomics in designing drug biomarkers to evaluate the susceptibility of the patient to a particular anticancer drug and provides an opportunity to markedly shift from one size fits for all approach to patient-oriented approach, personalized treatment and precision therapy (Figure 3) [15].

Over the last few years, various researchers investigated relationship between formation of drug induced DNA adduct levels detection in corresponds to cytotoxicity potential [45,46]. For instance, detection of platinum-DNA adduct using ELISA based trials in ovarian and testicular cancer patients who were treated cisplatin [47,48]. Chen et al. also reported increased levels of platinum-adduct formation when resistant cervical cancer cell lines were exposed to D-penicillamine in combination with cisplatin [49].

Furthermore, detection of Oxaplatin induced DNA adducts in colorectal cancer patients with a FOLFOX (combinational drug therapy containing Folinic acid, Fluorouracil, and Oxaliplatin) will help in designing and optimizing better treatment strategies for cancer patients. Upon treatment with FOLFAX, detected Oxaplatin-DNA adducts in PBMC were proportional to tumor reduction, which makes Drug-DNA adducts a potential biomarker in cancer treatments [50].

The nitrogen mustard compound cyclophosphamide is an alkylating agent used as anticancer agent. Cyclophosphamide requires to undergo metabolic activation by CYP2B6 enzyme to form phosphoramide mustard to formation of DNA adducts. There were increased DNA breaks and crosslinks were observed in peripheral mononuclear blood cells (PBCs) of ovarian cancer patients receiving combination of cyclophosphamide and carboplatin when compared to control healthy patients [51]. Increase in DNA breaks and crosslink were also correlated with increased therapeutic success. Similarly, In another study, HPLC-MS/MS analysis of blood cells of Fanconi anemia (FA) patients and non-FA cancer patients, there was increased DNA cross-link G-NOR-G were quantified upon cyclophosphamide-based therapy [52].

DNA adducts identification and quantification can be done by mass Spectrometry using SILAM (Stable Isotope-Labeled Adduct Mixture) and SRM (Selective Reaction Monitoring) through data acquisition and analysis. PR104A is an experimental anticancer agent which is a DNA-alkylating agent and hypoxia activated pro-drug, which produces cytotoxic activity through its metabolites Amine (PR104M) and Hydroxylamine (PR104H) which forms DNA adducts. These DNA adducts can works as biomarker to evaluate drug efficacy and explicates the cellular and molecular effects of PR104A. Using SILAM-SRM technique it was determined that adduct formation was increased 2.4-fold due to PR104H and PR104M which was also associated with 2.6-fold increase in cytotoxicity in HT-29 cells. The outcome of the study conveys DNA adduct levels are connected with drug potency and PR104A-derived DNA adducts play the role of biomarkers of efficacy [53].

Based on above case studies and discussion it can be summarized that detecting drug-DNA adduct is a very promising tool for predictive biomarker for development of precision medicine. Despite of the potential benefits in drug development there are still challenges in detection of DNA adducts because of their very low levels in total DNA pool make them very difficult to detect. Contempt to the potential applications, extraction, and quantification of adducts from various biological samples such as tissue homogenate, blood and urine are still very difficult to achieve with current available technologies. However, ongoing research and improving extraction methodologies can give positive outlook in the study of chemical induced adducts quantification. This challenge needs to be surmounted to exploit fully the potential of drug-DNA adducts as predictive biomarkers, which can be leveraged to provide personalized treatment in cancer chemotherapy.

#### 2.3.1. Detecting DNA Adducts in Oral Cells as a Potential Biomarkers for Detecting Lung Cancer Progression in Smokers

Study revealed the importance of DNA adducts in oral cells as potential biomarkers for the assessment of vulnerability of cigarette smokers to lung cancer [54]. Cigarette smokers are exposed to the highest risk of carcinogenesis, and this propels the need for biomarkers that would forewarn the impending threat, providing an opportunity for recourse to appropriate preventive measures. Traditionally, it was well established too, the tobacco carcinogenesis can be predicted by diagnosing and quantifying the urine and serum metabolites (Total nicotine equivalents) [55], Total 4-(methylnitrosamino)-1-(3-pyridyl)-1-butanol (NNAL), Phenanthrene tetraol (PheT) [56], 3-Hydroxyphenanthrene (3-OH-Phe) [57], S-Phenylmercapturic acid (SPMA) [58], 3-Hydroxypropylmercapturic acid (3-HPMA) [58], 3-Hydroxy-1-methylpropylmercapturic acid (HMPMA), Monohydroxybutylmercapturic acid (MHBMA) [59], F2-Isoprostanes (8-iso-PGF2α) [60] and Prostaglandin E2 metabolite (PGEM) [61] of the tobacco toxicants and carcinogens. Though this provides a holistic picture of exposure profile to tobacco to some extent and, in some cases, evaluate the risk to lung cancer it has inherent limitations associated with it. Metabolites in serum and urine serve as biomarkers of exposure scenario of the individual, but they would not yield critical information regarding DNA adduct burden and DNA damage parameters that induce mutations in cancer control genes such as KRAS and TP53. Furthermore, the mere presence of the metabolites does not proportionate with the extent of adduct burden owing to inter person variability of their ability to detoxify and repair DNA damage. To address this limitation, oral cell DNA adducts (specific to tobacco) are used as biomarkers to evaluate the vulnerability of the smokers to mutagenesis. Moreover, a strong correlation has been established between molecular aberrations in oral mucosal cells and bronchial cells due to tobacco smoking, which was evident in several studies that demonstrated promoter methylation patterns of p16 and FHIT genes and similar gene expression changes in specimens collected from both the oral (nasal and buccal) tissues and lungs from smokers [62,63,64]. When studies were performed using oral and salivary DNA to evaluate DNA adducts, a few adducts were identified, which were previously reported in lung DNA from smokers. These results convey that oral cells serve as a surrogate for lung cells in assessing and evaluating DNA adducts, obviating the need to isolate bronchial cells in risk assessment. Adding further, oral mucosa cells also offer the advantage of relatively simple to collect, which is contrary to bronchial brushings and sputum collection from the lungs that is impractical, difficult, and expensive [65].

#### 2.3.2. Glycated Hemoglobin, Protein Adduct—A Diagnostic Tool for Diabetes and a Classic Example of the Application of Adductomics

Significance of the adductomics would be deduced from the fact that the discovery of glycated Hb (HbA1c), a protein adduct, by Samuel Rahbar in the 1960s [66,67] paved the way of diagnosis of diabetes using HbA1c as a potential biomarker. The discovery of the Glycated Hemoglobin is widely hailed as a landmark event in the study of the non-enzymatic chemistry in biological systems, and it is probably the earliest application of adductomics in the health care system. In diabetic patients, the levels of glycated Hb (HbA1c) are high when compared with non-diabetic, and HbA1c, glycosylated hemoglobin, or glycohemoglobin (adduct), is formed by the non-enzymatic reaction of glucose with hemoglobin in the blood. HbA1c test measures average blood glucose over months, and it provides an indication of long-term blood glucose control, and this also serves as a regular monitoring tool if a person has been diagnosed with diabetes. The HbA1c test can interpret average blood glucose levels of approximately 6–8 weeks. Even though the test employs High-Performance Liquid Chromatography (HPLC) for quantifying HbA1c, the core principle of the test is vested in adductomics. Today, measurement of HbA1c is an established procedure in diabetic patients, and it is of great importance because HbA1c levels reflect the risk of developing diabetes-related complications. HbA1c, a first observed product of non-enzymatic glycation of proteins in 1960, accentuated advanced concepts of glycation/lipoxidation end products [68].

### 2.4. Adductomics in Preventive Healthcare or Adductomics in Predictive and Prognosis of Diseases

Tools that predict the probability of onset of disease equips us with strategies to prevent the disease, and this predictability is better served with adductomics, which demonstrates a new dimension in addressing some of the critical issues prevalent in the health sector. Currently, the world is facing tobacco smoking epidemic and it is emerged as one of the biggest public health threats decimating more than 8 million people a year around the world, and what’s more worrying is tobacco consumption kills up to half of its users. According to the World Health Organization (WHO), non-communicable diseases are the leading cause of deaths globally, and tobacco smoking is one of the foremost risk factors behind the emergence of non-communicable diseases. At least 17 types of human cancers, to name a few such as Small Cell Lung Cancer, Lung Adenocarcinoma, Acute Myeloid Leukemia, and Colorectal cancer, are associated with tobacco smoking is expected to cause death to 6 million people every year. One of the primary reason for tobacco smoke being one of the most lethal is because of its composition it contains several chemicals among which at least 60 substances are known carcinogens, and many of these damage DNA-inducing carcinogenesis. DNA damage is triggered by the reactive electrophiles that covalently bind with DNA, forming DNA adducts, and this covalently modified DNA is mis replicated during the DNA replication inducing prejudicial errors and undesirable mutations, setting the stage for mutagenesis [66]. Furthermore, elevated levels of tobacco induced DNA adducts were found in tissues like throat and lungs in smoker patient, surprisingly similar results were also found in organs which are not directly exposed in smoke like bladder when compared to control patients. Which provide possibilities of causing cancer through smoke is not limited to organs like lungs and throat [50,51].

DNA adducts are physical complexes formed with DNA by reactive chemical species interaction with DNA and detecting these adducts would serve as potential markers for determining the ’biologically effective dose’ for the carcinogens presence in tobacco smoke and can help better monitoring smokers health. Several studies revealed tobacco smoke exposure can potentially induce the formation of DNA adducts in in vivo studies, and they have shown positive correlation with carcinogenesis. In addition, detection of DNA adducts may also provide a comprehensive measurement of carcinogens exposure also in cancer risk assessment and prediction. Several clinical and epidemiologic studies have established the association between increased DNA adducts levels and the chances of tobacco-related cancers such as lung, head, neck, and bladder cancers [52,53]. While the DNA adduct profile provides exposure pictures, the DNA adduct burden assesses the risk of carcinogenesis. Induction of DNA adducts in blood lymphocytes is also perceived to be associated with the development of head and neck cancer. However, the dose-response relationship between smoking and DNA adducts in exposed organs is not fully characterized, and in fact, this relationship is complicated because of the inconsistencies in epidemiologic studies, and it is assumed genetic polymorphism (in metabolism of carcinogens (e.g., GSTP1) and DNA repair (e.g., XRCC1)) is the underlying reason. In the tumor cases at an early stage of tobacco carcinogenesis, induced by p53 mutations and DNA adducts, it was witnessed that levels of DNA adduct were correlated with somatic alterations (e.g., 3p21 LOH) [69].

### 2.5. Furan Toxicants and Adductomics

Furan is a ubiquitous agent which is very much used in the synthesis if various chemicals and pharmaceutical substances during the manufacturing process. Study provided new insights into furan induced hepato-carcinogenicity in rats, mediated through reactive metabolites, by forming adducts with Histone H2B [70]. Aberrant Histone modifications invoke unusual epigenetic alterations, deviant gene expression, and disturb nucleosome stability [71]. Furan is a rodent hepatogen-carcinogen prevalent in the environment as environmental pollutants emitted by cigarette smoking [72], gasoline and diesel-burning, and wood smoking, and they are also present in heat-processed foods as food contaminant [73]. Furan, being a non-reactive compound first need to be transformed into reactive electrophile, cis-2-butene-1,4-dial (BDA), which is catalyzed by CYP P450 2E1 [74] and BDA forms an electrophilic conjugation with glutathione molecule forming conjugated GSH-BDA complex. Thereafter, GSH-BDA reacted covalently with lysine residues in Histone protein, yielding GSH-BDA protein adduct. Traditionally it is presumed that histones are the target proteins for reactive electrophile carcinogens, and covalent modifications in histones are induced by chemical carcinogens resulting in abnormal epigenetic makeup and skewed transcriptional activity [75,76,77]. Histone modifications with respect to time and dose were also carried out in furan treated rats, for the first time, the presence of covalently modified Histone-carcinogen complexes in-vivo were observed. In addition. Detection of Tryptic peptide using LC-HRMS (liquid chromatography—high resolution mass spectrometry) reveals that cross link between GSH-BDA and lysine 107 of Histone H2B in isolated liver cells found to be covalent bond. These adducts were detected even at exposure to low dose (0.92 mg/kg body weight/day) for 90 days treatment and triggers induction of carcinogenesis. The incorporation of GSH-BDA into the tryptic peptide is evident from the mass increment of 355.0838 amu, the hallmark of the reactive electrophile, GSH-BDA. This study widens the scope of understanding of cancer and facilitates the development of exposure-specific biomarkers by employing adductomics that signals hazardous chemical exposure and risk of carcinogenesis associated with it [78].

### 2.6. Adductomics in Designing Tools for Food Safety Assessment

The principle of adductomics is being explored currently in crafting tools/methods/technologies/techniques for detecting toxic chemical agents, which are detrimental to living organisms and the environment. Recently, the Institute of Advanced Study in Science and Technology (IASST), Guwahati, has developed an electrochemical sensing platform for detecting mutagenic compounds N-nitrosodimethylamine (NDMA) and N-nitrosodiethanolamine (NDEA) [79]. This remarkable achievement was fueled by designing a modified and advanced electrode using immobilized carbon nanomaterials (carbon dots) in DNA. NDMA and NDEA are potent carcinogens detected in food products like cured meat, bacon, some cheese, fish, and low-fat milk and the meat [75,80]. These toxic carcinogens are formed during the foods were processed by smoking, curing, salting, or processed with potassium and sodium nitrate to reduce botulism [81,82]. Moreover, U.S Environmental Protection Agency (US EPA) has reported that N-nitrosamines are classified as possible carcinogens to humans, which makes important to identify them in food samples [83,84]. With the changing food habits in this contemporary era where processed food items are gaining attention, it has become inevitable to regulate their human consumption by conducting safety assessments [85]. N-Nitrosamines are generally detected by spectral methods chromatography and electrochemistry with detection limit mostly in µM [66,86,87]. The electrochemical method is a very low cost, precision, simple, fast, with very lower detection limits makes it unique and scalable technique. This newly developed electrode applies the principle of electrochemistry to identify N-Nitrosamines in the food samples, besides the fact that the underlying mechanism involves adductomics. Among the four base pairs (Adenine, Guanine, Thymine, and Cytosine) of the DNA, Guanine (G) is electrochemically active, and this basic fact is used to design the tool, which could diagnose NDMA and NDEA. NDMA modifies guanine to 6-O-methyl guanine or 7-methyl guanine, and NDEA changes guanine to 8-oxoguanine forming DNA adducts, which are electrochemically active, more active than Guanine base alone. These modified Guanine bases and DNA adducts causes increase peak current in the electrochemical set-up that indicates detection of N-Nitrosamines in samples. It pitches new insights into the benefits of adductomics and a combination of adductomics with other principles/technologies, opening new vistas for developing diagnostic tools and methods [88,89].

#### 2.6.1. Applications of Adductomics in the Verification of Chemical Warfare Agents in Biological Specimens

Chemical warfare (CW) is specialized, and most brutal methods of mass destruction created by humans, and it’s devastating potential is placed at equal footing with biological and nuclear warfare. CW agents deliver either incapacitating or lethal effects on humans, even in minuscule doses. CW agents are highly reactive synthetic toxic chemicals that can be dispersed in physical forms (gas, liquid, aerosol or adsorbed to particles). Even though there are thousands of toxic substances, only a few of them fit into the criteria of CW agents (CWA) because characteristics like imperceptibility to senses, high toxicity, persistency and rapidity of action after dissemination, these are only listed as scheduled chemicals in the Chemical Weapons Convention (CWC), a global inter-governmental convention which exclusively deals with chemical weapons. According to the CWC, CWAs are defined as “toxic chemicals and their precursors, munitions and devices, and any equipment specifically designed for use directly connected with such weapons.” Incidents erupted regarding the use of CWAs in Syria, Malaysia, and the UK recently demonstrates the continuing threat of chemical warfare agents in the modern world [90,91,92,93]. Identification of the exposed chemicals is very much important to detect and prevent associated adverse health effects. Some of the toxicities can be detected by understanding covalent adducts of proteins and DNA formed after exposure to CWA and these can work as potential biomarkers for exposure assessment. Albumin and hemoglobin, the most abundant proteins in the blood, acts as readily available scavengers for many reactive chemicals, and adducts formed by reactive chemicals can serve as outstanding diagnostic candidates to determine the type of chemical exposure and adducts resulting from the nucleophilic interactions with blood proteins are valuable in the development of diagnostic markers. In human’s proteins, serum albumin, and hemoglobin may carry these adducts longer time even after the exposure is ceased. By employing adductomics tools, studying adducts facilitates diagnosing the type and nature of chemical agents exposed, assisting in extending adequate treatment to nullify the ill effects, and recognizing the biological mechanisms [94]. Speedy advancement in analytical techniques such as mass spectrometry, which acquired greater resolution over a period, generated high-quality data that to analyze DNA adducts of CWAs. This placed emerging ‘adductomics’ at equal footing with other “omics” technologies serving as one of the most potent bio-analytical tools for verifying CWAs exposure.

#### 2.6.2. Environmental Adductomics—Linking DNA Adducts with Embryo Aberration in Baltic Amphipods

Environmental adductomics added a new dimension in recognizing the role of environmental stressors (pollution and climate change) on humans and wildlife health. Prior studies deduced the relationship between environmental contaminants and reproductive disorders/embryo aberrations signifying the role of detecting embryo aberrations in environmental health assessment. Further, several aberration types documented in amphipods were connected to exposure to specific toxicants such as polycyclic aromatic hydrocarbons (PAHs), polychlorinated biphenyls (PCBs), and heavy metals present in ambient sediments [95]. Currently, Swedish National Marine Monitoring Program (SNMMP) is expending the detection of embryo aberrations in amphipods as a biological indicator of detrimental effects of pollutants, and relative dominance of the aberrant embryos is a potential supporting indicator under the category Descriptor 8 in the Marine Strategy Framework Directive (MSFD) [14]. Moving a step ahead, adductomics connected embryo aberrations in amphipod Monoporeia affinis with environmental contaminants assessed using adducts as biomarker. In a study M. affinis (Amphipods) from the Baltic Sea, was analyzed using HRMS/MS to identify DNA adducts. DNA nucleoside adducts were identified in gravid females, which correlated with the embryo aberrations in offspring 8 out of 23 putative nucleoside adducts were observed in both females and embryos were identified structurally utilizing accurate HRMS data. Partial Least Squares Regression (PLSR) modeling identified three adducts which are DNA (5-methyl-2′-deoxycytidine), DNA (N6-methyl-2′-deoxyadenosine) and one unidentified structural nucleoside adduct. A research study summarized with high classification accuracy (84%) that the environmental contaminants are associated with increased frequency of the embryo aberrations in species extant in the wild. Probably it was the first study that applied adductomics in field-collected animals to decipher contaminant driven malformation in the embryo, inducing reproductive toxicity [96]. This omics approach can be replicated to other diverse species, equipping us with a new environmental health assessment tool. Moreover, it adds a new dimension in assessing environmental pollutants; it is a marked deviation from the traditional approach, where environment pollutants, in samples (water samples/air samples/land samples) collected from the environment, are detected and appraised using the chemical analysis/chemical methods.

### 2.7. DNA Adductomics—A Confirmatory Tool in the Assessment of DNA Damage

Genetic toxicity assessment holds a high priority in safety risk management while developing new chemical compounds, and it does so by evaluating carcinogenicity and mutagenicity of a particular chemical, thereby assisting in hazard identification and risk characterization of chemical agents [97,98]. Traditionally, genotoxicity, mutagenicity and carcinogenicity potentials of a chemical is evaluated by using Ames assay, chromosomal assay, and micronucleus assays [99,100]. However, the challenging aspect with those in-vitro methods is high rates of false-positive outcomes that demand the need to develop novel methodologies and pathway-based understanding of toxicity, which could provide a more accurate picture of DNA damage that could directly detect DNA modifications and DNA damage at molecular level [101,102,103]. Here, DNA adductomics turns out to be a potential candidate methodology that could comprehensively investigate DNA damage through direct molecular detection by identifying and quantifying DNA adducts [104,105,106]. The Micronucleus test is one of the widely used in-vitro tests to assess DNA damage, but it is now supplemented by DNA adductomics to nullify the error due to false-positive outcomes from the test, which signify the confirmatory role of DNA adductomics in other in-vitro assessments of genotoxicity [107,108].

### 2.8. Adductomics: Role in the Human Exposome Project (OR) EXPOSOMICS Project

The Human Exposome Project or EXPOSOMICS Project is a European Union funded project that provides an assessment of high priority environmental pollutants using the Exposome approach, which includes internal and external Exposome components [109,110]. It serves as a toolbox for assessing and addressing the impact of the environment on health. Exposome is a measure of summation of exposure that individual experiences in his lifetime (covers both internal and external components) and evaluates its association with pathogenesis [111]. Decoding Exposome facilitates in addressing public health challenges and reducing the disease burden [112]. Exposome application assumes that individuals are much more than the genes, and it collates the non-genetic influence of the environment on health and disease. Exposome covers all dimensions of exposures that come from the environment, such as diets, lifestyles, infections, stress, drugs, radiation, pollution, and behaviors, and covers internal processes such as lipid peroxidation, inflammation, and oxidative damage [113]. Exposome assists in better comprehending the etiology of chronic diseases because it is generally accepted that several chronic diseases result from the complex interplay of chemical exposures, physical stresses, and genetic influence, and Exposome investigates the environment counterpart. The internal component of the Exposome requires measuring biomarkers of internal exposure, and it requires omics technologies; adductomics is one among them, making it imperative for the Exposome approach [110]. Adductomics, an omics tool, measure the adduct levels in the individual that are generated by the reaction of the external and internal reactive electrophiles with biological macromolecules, providing an overview of exposure sketch in the individual.

## 3. Other Applications

In addition to the above, other applications of adductomics are presented in the Table 1 below along with the titles of the publication along with novel applications of adductomics:

## 4. Current Challenges and Future Perspectives

Advancements in diagnostic tools and the emergence of new technologies gave rise to the applications of adductomics. However, still there are challenges which are needed to be addressed to exploit fully the potential of adductomics in toxicological and environmental assessment of chemicals. Though data-dependent and data-independent acquisition methods (in untargeted adductomics “omics technologies”) were developed to simultaneously screen multiple adducts, obstacles in the data processing need to be addressed get a precise picture of toxicants [104]. The low frequency of DNA adducts in the sample pool also presents a serious challenge to the current software in the realistic assessment that uses common data acquisition methods. This demand for the continuance of the data processing software and improvements in the algorithms to detect adducts, even in low concentrations that grant critical to comprehend pathogenesis [143]. There is a scope of improvements in sample preparation and clean up when it comes to the detection of hydrophilic adducts. In addition, incomplete enzyme hydrolysis fails to generate and observe certain types of DNA adducts, demanding a comprehensive assessment of the merits and demerits of several enzymes for DNA hydrolysis and their optimal utilization. In adducts whose molecular weights are below 70 KDa there are few probable structures, and their identification is not troublesome, but in adducts with greater molecular weights, their characterization is extremely difficult because of the widened possibilities and amplified permutations; this is the issue of concern even though we might make accurate mass measurements and produce ion-fragmentation spectra. This impairment can be overcoming by crafting a database of adducts that would provide ready information regarding the adducts; unfortunately, there is no specific database for adductomics even though everyday hundreds of DNA adducts are being characterized globally, creating such a database entails thorough literature search of molecular formulas of already characterized adducts. Fragmentation spectra produced from both ion trap and quadrupole-type fragmentation at the MS2 and MS3 levels demonstrated at various collision energies would become handy if compiled and integrated into database. At present, databases like Search for Species Data by Molecular Weight provided by NIST (National Institute of Standards and Technologies) [146], UNIMOD [147], Human Metabolome Database [142], Toxic Exposome Database [143], Exposome-Explorer Database [144] find applicability in adductomics. But the databases mentioned above are not specific to adductomics, which requires the creation of a dedicated database that can facilitate easy identification of unknown adducts. There is a need to develop more robust and simple technology to further improve in sample collection, as suggested above more focused approach on noninvasive liquid sampling, optimization of sample preparation methods which can give precise and reproducible results is required. Current analytical techniques are very time consuming and expensive to run the samples, further development in cost effective analytical techniques can further potentiate applications of adductomics in biomedical research. Despite the limitations mentioned above pace of technological advancements gives us optimism that limitations that are challenging the adductomics from reaching its fullest potential will be addressed soon with the help growing scientific advancements in sample processing data collections and data analysis tools.

## Figures and Tables

**Table 1:** Summary of the various applications of adductomics in biomedical research in various research articles published in recent times.

Research Areas and Application of Adductomics	Significant Outcomes of Adductomics Technology Application in Various Studies
Paraoxonase-1 and Early-Life Environmental Exposures—Application of adductomics in biomonitoring of organophosphates exposure using OP-adducted biomarker proteins [114]	Children and infants are vulnerable to Organophosphates, which are widely used pesticides in agriculture, because detoxification mechanisms are not developed to their fullest potential, that only happens several years after birth. Paraoxonase-1 (PON1) is a detoxification enzyme that neutralizes the toxic effects of organophosphates, and the status of PON1 in the mother determines the level of protection in the child against the adverse effects of organophosphates. Though PON1 status gives an account of the susceptibility to the organophosphates, analyzing OP-adducted biomarker proteins becomes handy in biomonitoring of the exposure trends. Moreover, using adductomics instead of enzymatic activity assays (cholinesterase enzymatic inhibition assay) also offers multiple fold of advantages, and the use of dried blood spots would suffice to monitor the pesticides in infants and mothers.
Cys34 blood adductomics method [115]	Cys 34 residue in Human Serum Albumin is highly reactive to several reactive electrophiles [116], and this non targeted approach is used to distinguish: smokers from non-smokers [117] and users of smoky coal from smokeless coal [118]. Cys 34 residues are also implicated in several adducts that form after exposure to multitude of hazardous exogenous agents.
Organophosphate-adducted serine esterase—Classical Biomarker of pesticide exposure [119]	Organophosphate-adducted serine esterases are among the most widely studied and known protein adducts employed in biomonitoring of pesticide exposure in humans. There is a growing interest protein biomarker-based identification of pesticide exposure because protein adducts have longer half-lives, and proteins are only modified by the active parent molecule but not by their breakdown products or metabolites. The cited reasons above ensure a realistic assessment of pesticide exposure with adductomics when compared with other methods of assessment [119].
DNA adductomics application to soil bacterium Sphingobium sp. strain KK2 [120]	This was the first study to report DNA damage in bacteria and paved the path for the use of microbes in toxicity studies using biomarker adducts. Sphingomonad bacteria are renowned for their existence in polluted ambiance, especially in the areas contaminated with hazardous hydrocarbon pollutants [121,122]. Acrolein is a biocide used in the hydraulic fracturing process of hydrocarbon extraction, and bacteria are exposed to this pollutant. Later the DNA was extracted and analyzed for DNA adducts using positive ionization electron spray LC-MS in pre-set reaction monitoring mode transmitting the [M + H] +> [M + H−16] + transition over 100 transitions. Two exposure-specific adducts: 3-(2′-deoxyribosyl)-5,6,7,8-tetrahydro-6-hydroxyl PdG and 8-hydroxy-pyrimido [1,2-a]-purine-(3H)-one PdG (6-and 8-hydroxy-PdG) were selected. These 6-and 8-hydroxy-PdG putative adducts were specific to Acrolein, and they serve as potential biomarkers of Acrolein contamination in the environment. This study demonstrated the application of DNA adductomics using soil microbes to assess environmental contamination and develop compound-specific biomarkers for prognosis of pollutants [120].
Comparing mutagenic activity of the benzene metabolites: hydroquinone (HQ) and para-benzoquinone (p-BQ) using supF forward mutation assay [123]	Benzene is a human leukemogenic and rodent carcinogen that is omnipresent in the atmosphere as a hazardous occupational chemical. Though the precise mechanism underneath the mutagenicity of benzene is not deciphered, it was assumed that benzene-DNA adducts play a crucial role in mutagenesis. Benzene is a neutral molecule, and it is catalyzed in the body to generate reactive electrophiles, and these reactive metabolites intercalate with DNA and proteins invoking detrimental effects. In this in-vitro study Hydroquinone [HQ] and Para-benzoquinone [p-BQ], from among the pool of electrophiles generated from benzene, react with DNA forming carcinogenic adducts (dGp adducts); p-BQ yielded four adducts while HQ yielded only one DNA adduct. Mutagenicity of these reactive metabolites is investigated using the supF forward mutation assay and screened using an indicator bacterium. Upon comparing the mutagenic potential of both the metabolites, it was revealed that 5–20 mM p-BQ treatment resulted in a 12–40-fold increase in mutation rate, whereas 5–20 mM HQ treatment resulted in an 8–26-fold increase in mutation rate, which signifies the greater mutagenic potential of the former that the later. The study revealed that dGp adducts formed by the benzene metabolites are instrumental in mutagenicity and myelotoxicity of benzene [123,124,125,126].
Adenine-Colibactin adduct—Biomarker for Colonorectal Cancer [127]	Colibactin, polyketide/nonribosomal peptide produced, is produced by Escherichia coli that encompass biosynthetic gene islands called pks. Colibactin is a genotoxic secondary metabolite implicated in Colorectal cancer (CRC), which induces mutagenesis by forming, adducts culminating in the breaking of double-stranded DNA. Colibactin contains a reactive cyclopropane ring that reacts with DNA (adenine) to generate adenine-Colibactin adducts, and these adducts serve as biomarkers for pks + E. coli exposure, providing a forecast of the genesis of colorectal cancer [127].
Adductomics Research Programme [128]	It is a flagship program of the National Cancer Institute (Division of Cancer Prevention) that aims at applying the principles of the adductomics to design strategies for cancer screening and prevention by identifying molecular signatures/biomarkers that bear a testament for risk exposure. Moreover, the program also laid emphasis on amalgamating adducts signatures with other molecular signatures (DNA repair defects and capacity to repair, genetic aberrations and alterations, imaging, role of non-coding RNAs in cancer risk) to present the true situation in the cell, which facilitates early cancer identification and risk prevention. The program also aids projects investigating the potential roles of adducts in gene-environment interaction research (GxE) and cancer etiology.
Urinary DNA adductomics [129]	DNA adductomics is an inalienable part of the exposome approach, and invasive sample collection methods (blood or tissue samples) are generally adopted to isolate the required DNA for sensitive analysis of DNA adducts. However, invasive tissue sampling to obtain sufficient DNA poses IRB and logistical challenges, particularly when investigating vulnerable populations. This study provided a novel approach of adductomics in biomonitoring environmental exposures in a non-invasive manner using a urine sample, easing the exposome’s evaluation across the life-course, which reflects the totality of body burden of adducts amassed overtime.
Cys34 Adductomics Links Colorectal Cancer with the Gut Microbiota and Redox Biology [130]	It is well very well known that chronic colonic inflammation is implicated in inflammatory bowel disease and colorectal cancer mediated through reactive oxygen species (ROS) and reactive carbon species (RCS) that alters biomolecules (proteins and DNA) and modulates redox signaling pathways. ROS and RCS, despite holding huge potential of predicting the risk of colorectal cancer, cannot be measured in-vivo and to overwhelm this barrier, untargeted adductomics approach was used to identify reactive electrophile products of human or microbial metabolism by monitoring adducts of those species that react with hemoglobin and Human Serum Albumin (HAS). In this study, the adductomics pipeline was developed to investigate amendments at the highly nucleophilic Cys34 residue in HAS and the rationale for the selection of Cys34 in HAS was for its involvement in scavenging reactive electrophiles. Using untargeted adductomics, the study was performed to diagnose adducts in pre-diagnostic serum collected from the EPIC Italy cohort. For the study, seven Cys34 adducts which are associated with progression of colorectal cancer, and body mass indexes (BMI) are carefully chosen as risk factors. 5 out of 7 adducts were identified predominant in colorectal cancer cases when compared with controls. In those five adducts, two of them were modified by methanethiol (a microbial–human cometabolite), and crotonaldehyde (a product of lipid peroxidation). This manifests inflammation, a precursor step to colorectal cancer, caused by gut microbiome infiltration into intestinal mucosa. It also demonstrated that selected adducts and BMI would serve as potential casual factors when compared to other variables such as processed meat consumption and smoking that were associated with progression of colorectal cancer previously. The study exposed the role of Cys34 adducts of HSA taking the role as biomarker for calculating the chances of onset of colorectal cancer [131,132,133,134,135].
Biomarkers in epidemiology studies [17]	Several biomarkers (carcinogen-DNA adducts) pertinent to tumor initiation and progression are also currently under development. In addition to DNA, albumin also forms adduct aflatoxin B1 which is human carcinogen which were successfully explored as biomarkers in molecular epidemiology studies. Other examples such as Benzo(a)pyrene (BP)-like DNA adduct biomarker gives clues regarding breast cancer etiology and Putative lipid peroxidation-related DNA adducts role in forecasting pancreatic cancer is also explored. The DNA adducts formed from specific chemicals are used as biomarkers in molecular epidemiology and cancer prevention studies. Benzene exposure is resulting in the induction of oxygen and carbonyl species adducts and Cys34 disulfides of small thiols in HSA, which serve as adductomics signatures offering significant insights into cancer [135,136,137,138,139,140,141].
HELIX project and adductomics [140]	The HELIX project investigates the impact of early life exposure of chemical and physical agents in existing birth cohorts, while EXPOSOMICS project pays emphasis on the assessment of the effect of air pollution studied in an adult and child populations [109]. Both these projects involve two components: exposure monitoring using technology support and investigating biomarkers associated with exposures using a multiomics approach, and the second component include metabolome, proteome, transcriptome, epigenome and adductome profiling [110].
Protein adductomics and its applications [135]	Biomonitoring of protein-adducts formed due to carcinogens is one of the superior and effective substitutes for the detection of DNA adducts for risk assessment owing to the abundance of proteins, easy detection, and stable adducts for longer duration [142,143,144]. Several proteins, such as albumin, hemoglobin, glutathione, and histones in the body, form adducts with reactive chemicals. Studying protein adducts also facilitates evaluating the quality of the environment as proteins in several biological systems possess nucleophilic reactive sites that easily form adducts with toxicants, and the extent and type of adduct provide us vital information pertinent to the quantity and category of pollutants prevalent in the environment. This is substantiated by the fact that some of the common industrial pollutants such as benzene, isocyanates, naphthalene, aromatic amines form adducts with albumin. Albumin, being the most abundant protein, forms adduct with so many very well-established therapeutic drugs or their reactive metabolites such as acetylsalicylic acid, non-steroidal anti-inflammatory drugs, acetaminophen, β-lactam antibiotics, antiretroviral therapy drugs and chemotherapeutic agents. Identifying and characterizing adduct structures formed by Alb with drug metabolites play a key role in understanding the generation of reactive metabolites and assisting in predicting idiosyncratic drug reactions toxicities [134,135]. The Alb-drug metabolite adducts are included in a series of screening tools and technologies in assessing potential toxicities of drugs, and it also aids in calculating the biologically effective dosage of drugs and developing personalized, and precision treatment approaches, which minimizes adverse effects of drugs. Several proteins adduct are becoming beneficial in predicting early and late biological effects of several chemicals and toxicants [145].
